# Characterization of Tat Antibody Responses in Chinese Individuals Infected with HIV-1

**DOI:** 10.1371/journal.pone.0060825

**Published:** 2013-04-02

**Authors:** Qiuli Chen, Lan Li, Wenting Liao, Hongwei Zhang, Jinhong Wang, Bo Sheng, Huaqun Zhang, Xiaojie Huang, Yingying Ding, Tong Zhang, Jie Cao, Hao Wu, Wei Pan

**Affiliations:** 1 Department of Microbiology, Second Military Medical University, Shanghai, China; 2 Center for Infectious Diseases, Beijing You’an Hospital, Beijing, China; 3 College of High Altitude Military Medicine, Third Military Medical University, Chongqing, China; University of South Carolina School of Medicine, United States of America

## Abstract

HIV-1 Tat is an important regulatory protein involved in AIDS pathogenesis. However, the immunoprofiles of anti-Tat responses remain unclear. We analysed the immunoprofiles of the anti-Tat antibody responses and the neutralizing activities. Out of 326 HIV-1-seropositive individuals, 12.9% were positive for anti-Tat antibodies. We found six different immunological profiles of anti-Tat antibody responses: full-potential response, combined response, N-specific response, C-specific response, full-length Tat-specific response and Tat-related response. These responses represent two types of anti-Tat responses: the major complete response and the alternative C-prone response. A Tat-neutralizing activity is significantly higher in anti-Tat-seropositive samples than anti-Tat-negative or healthy blood-donor samples, and significantly correlates with the anti-Tat reactivities. The data here could contribute to a better understanding of the significance of anti-Tat responses in preventing HIV pathogenesis and could be useful for designing more effective vaccines in the future.

## Introduction

As one of the six accessory proteins of HIV-1, Tat is synthesized during both the early and late stages of viral replication and is critical for these processes. The HIV-1 Tat protein is encoded by two exons and can be between 86 and 101 amino acids (aa) in length, depending on the specific viral strain. The Tat protein can be divided into six functional domains[1], [2]: (1) the N-terminal acidic aa region (aa 1-21), which has been linked to Tat immunosuppressive activity[3], [4], [5], [6]; (2) the cysteine-rich region (aa 22–37), which is responsible for transactivation of transcription; (3) the “core” region (aa 38–48), which is highly conserved; (4) the basic region (aa 49–57), which recognizes the transactivation response element (TAR) [7]and plays important roles in both the nuclear localization of Tat [8] and the entry of extracellular Tat into bystander cells[9]; (5) the glutamine-rich region (aa 60–72), which has the highest rate of sequence variation; and (6) the C-terminal region (aa 72–86 or 72–101), which is encoded by the second exon and contains the “RGD” motif that allows Tat to bind integrin [10], [11]. Furthermore, Tat is actively released from HIV-1-infected cells [10], [11] and acts as an extra-cellular toxin [12], which plays a crucial role in HIV-1 pathogenesis, including development of HIV-associated dementia, HIV-related opportunistic infections and Kaposi's sarcoma.

Previous studies have shown that approximately 20% of infected individuals produce detectable amounts of Tat-specific antibodies, and the presence of anti-Tat antibodies is strongly correlated with slower disease progression and that no AIDS events were observed in persistently anti-Tat-seropositive subjects[13], [14], [15], [16], [17]. These results strongly suggest that Tat is a promising target for the development of both preventive and therapeutic vaccines [18], [19]. However, several contrary results were also reported[20], [21], [22], and the detailed host anti-Tat antibody responses remains unclear. In this study, we performed anti-Tat immunoprofile analysis in 326 Chinese individuals infected with HIV-1 and defined six immunological profiles of anti-Tat antibodies responses. Our findings provide a novel source of information with respect to anti-Tat responses and Tat-neutralizing potential that should be very important for understanding the role of this response in the prevention of HIV pathogenesis and vaccine design.

## Materials and Methods

### Ethics statement

All aspects of the study were approved by the Ethics Committee of Beijing You An Hospital,Capital Medical University, China. Written informed consent was obtained from all participants in the study.

### Vectors, bacterial strain and reagents

The prokaryotic expression plasmid pPEPTIDE2, as well as the two *E. coli* host strains BL21(DE3) and DH5α, were purchased from Novagen (Germany). A mouse monoclonal antibody that recognizes the N-terminus of native and recombinant HIV-1 Tat (strain HXB2) was purchased from United States Biological. HRP-LD5 consists of HRP conjugated to LD5, which is a novel evolved immunoglobulin-binding molecule (NEIBM) with a characteristic structure of consisting of alternating Finegoldia magna protein L B3 and staphylococcal protein A D domains; this structure creates synergistic double binding sites for the VH3 and Vk regions of Fab as well as to IgG Fc [23]. HRP-LD5 shows high binding affinity for IgM, IgG and IgA [24].

### Clinical samples

Clinical samples for this study were collected from the AIDS high-risk Cohort at YouAn Hospital in Beijing, China. Informed consent was obtained from each of the participants prior to blood collection. Clinical information for each of the 326 samples was also recorded (Table S1). The cohort contained of 252 males (mean age = 33.6, SD = 8.5) and 74 females (mean age = 38.4, SD = 6.8). Mean CD4 counts/μl for the males and females were 437.4 (SD = 150.9) and 340 (SD = 283), respectively. The seropositive status of the participants was confirmed using ELISA (Diagnostic Kit for Antibody to HIV (ELISA), Shanghai Kehua Bio-Engineering Co., LTD., China) and Western blotting (HIV Blot 2.2 WB, MP Biomedicals Asia Pacific Pte. Ltd., Singapore). Control samples were obtained from 100 healthy blood donors who were confirmed to be HIV seronegative. All samples were stored at −80°C in 1.5 ml aliquots.

### Synthesis of the Tat N terminus

The peptide HIV-1 HXB2 Tat 1–21 aa (sTat1–21) was produced using solid-phase synthesis by Temple University (Philadelphia, USA).

### Expression and purification of recombinant full-length Tat and six Tat fusion peptides

**Figure 1:**
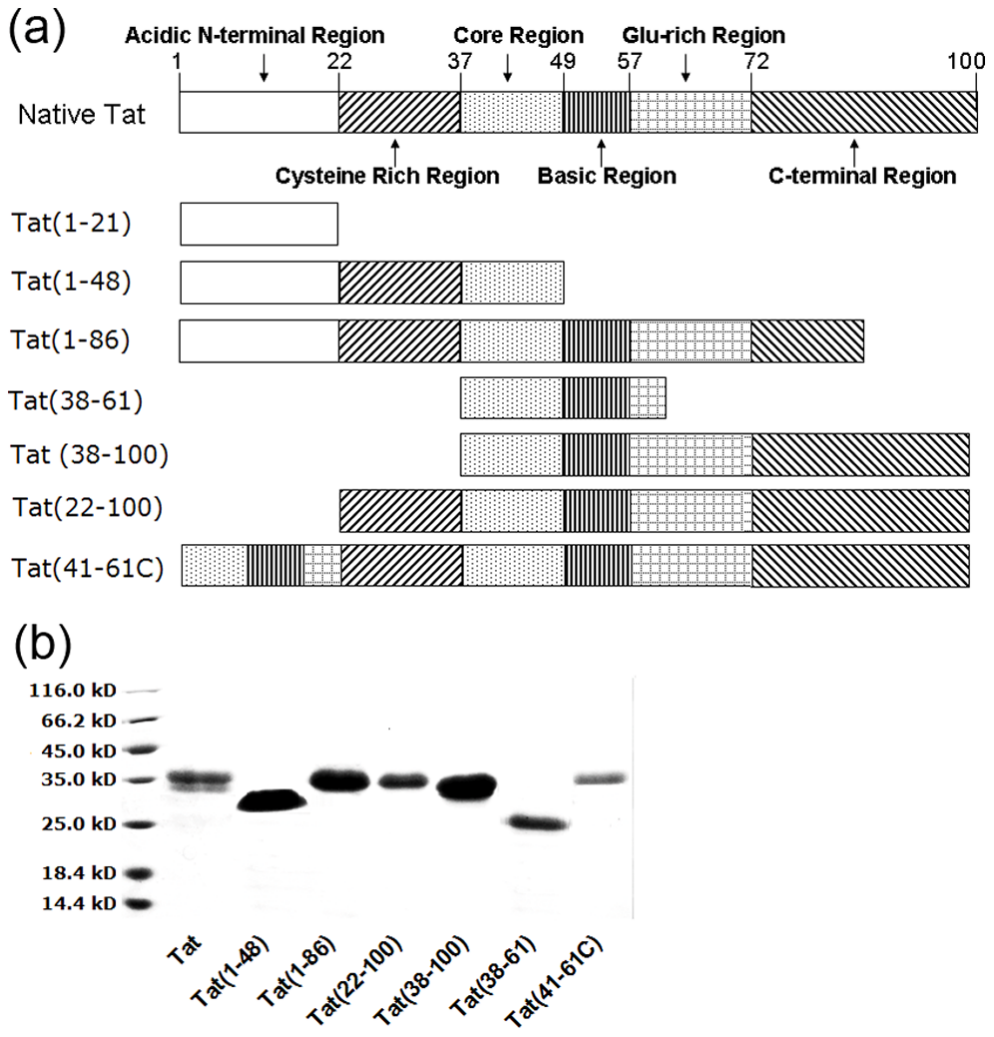
Design, expression and purification of Tat peptides. (a) Design of the seven analytic Tat peptides. (b) Expression and purification of recombinant full-length Tat protein and the six analytic Tat peptides. Recombinant full-length Tat and six analytic Tat peptides were induced by IPTG, and the relative molecular weight (MW) of the expressed products were as follows: 34,540 for Tat, 28,710 for Tat(1–48), 32,890 for Tat(1–86), 32,230 for Tat(22–100), 30,470 for Tat(38–100), 20,670 for Tat(38–61) and 36,850 for Tat(41–61C). The seven expressed products were purified by Ni-NTA column affinity chromatography and SP Sepharose fast flow ion-exchange chromatography.

The full-length Tat sequence from the HXB2 strain was obtained as described previously [25]. Full-length Tat, Tat(1–48), Tat(1–86), Tat(22–100), Tat(38–100) and Tat(38–61) were amplified by PCR and T/A-cloned into the pMD18-T vector (Takara). The primer pairs U41/D41–61C and UC/D100P were used to obtain Tat(41–61) and Tat(22–100), respectively, using a recombinant Tat plasmid as template. Tat(41–61C) was amplified by overlapping PCR using the primer pair Upet/Dpet with Tat(41–61) and Tat(22–100) as templates (Table S2, Fig. 1). Following cloning, verified DNA sequences were inserted into the prokaryotic expression vector pPEPTIDE2 at the AseI and BamHI sites. The seven plasmids were constructed as described above and transformed into *E.coli* BL21(DE3). They were induced and purified by Ni-NTA column affinity chromatography, as previously described [25], [26]. The purified proteins were immediately lyophilized and stored at −70°C until assayed.

### Indirect ELISA of antibodies against full-length Tat and Tat functional domain peptides

We used indirect ELISA to quantify antigen-specific antibody levels in the plasma samples using the recombinant Tat and six Tat peptides, largely as described previously [24], [25]. Briefly, immunoassay plates (Nunc, Rochester, NY, USA) were coated with 1.0 μg of full-length Tat, Tat(1–48), Tat(1–86), Tat(22–100), Tat(38–100), Tat(38–61) or Tat(41–61C) recombinant proteins and sTat1-21 in 50 mM carbonate buffer (pH 9.6) and incubated at 37°C for 3 h. The plates were blocked for 1 h at 37°C with 200 µl of 4% BSA prepared in PBS-Tween 20. Next, 100 μl of a 10-fold dilution of the plasma sample were added to appropriate wells. The plates was then placed in a 37°C incubator for 1 h. After washing four times with the wash buffer (50 mM Tris, pH 8.0; 100 mM NaCl; 0.2% Tween 20), 100 μl of a 1,000-fold dilution of HRP-LD5 (1 mg/ml) was added to the strip and incubated for 45 min at 37°C. The plates were developed by the addition of 3,3',5,5'-tetramethylbenzidine and absorbance at 450 nm was read using an ELISA Reader. Coated pPEPTIDE2 protein and samples obtained from healthy blood donors were included as negative controls. Samples showing absorbances above the mean value of the control group plus 3 SD (0.06 + 0.14) were considered to be positive for Tat-reactive antibodies.

### Tat-neutralization assay

The neutralizing potential of the anti-sera was evaluated by measuring their ability to inhibit the transactivation activity of native Tat (HXB2 strain) using a HEK293T cell line transfected with a plasmid encoding for the LTR of HIV-1 and secreted alkaline phosphatase (pLTR-SEAP) (kindly provided by Dr. Udaykumar Ranga) as described previously [25], [27] with minor modifications.

Briefly, 96-well plates were coated with 100 µl of pPEPTIDE2 (5 µg/ml in carbonate buffer (pH 9.6) and incubated at 37°C for 3 h. The plates were blocked for 1 h at 37°C with 200 µl of 4% BSA prepared in PBS/Tween 20. The plates were then incubated with 100 µl per well of 50-fold dilution of the HIV+Tat+, HIV+Tat− and HIV− plasma samples in DMEM for 2 h at 37°C to deplete the non-specific antibody. Then the depleted plasma were incubated with 500 ng of Tat protein at 37°C for 30 min. The samples were then added to appropriate wells containing cells and incubated for 3 h. The supernatant was removed and 200 µl of complete medium was added to each well. The plates were incubated for 48 h and the levels of secreted alkaline phosphate (SEAP) were estimated at 48 h using a colorimetric assay (Toyobo). As a control, 96-well plates were coated with 100 µl of full-length Tat protein (5 µg/ml in carbonate buffer (pH 9.6) or protein G to deplete the Tat-specific antibody or IgG fraction of the plasma. Then the depleted plasma were used for the inhibition assay. As a positive control for Tat neutralization, we used an IgG1 monoclonal antibody, which was raised in-house against B-Tat; this antibody recognizes the N-terminal 20 amino acid residues of Tat and blocks extracellular-Tat with high efficiency.

### Statistical analyses

Statistical analyses were performed using SPSS 17.0 and SAS 9.3. All experiments were performed three times, and the values obtained from three replicate samples were averaged for each experiment. Data are presented as the median and quartiles. Statistical significance was tested using Nemenyi or Wilcoxon non-parametric test. Differences between measurements were considered to be significant at p-values of less than 0.05. The correlation was assessed by Spearman correlation coefficient.

## Results

### Purification and characterization of recombinant Tat protein

We designed a set of recombinant antigens carrying different functional domains: Tat(1–48), Tat(1–86), Tat(22–100), Tat(38–100), Tat(38–61), Tat(41–61C) and sTat1-21 (Fig. 1a). These proteins were expressed in *E. coli* and then purified. Purified Tat proteins were relatively pure and largely free of contaminating bacterial protein, as assessed by SDS-PAGE electrophoresis (Fig. 1b).

### Anti-Tat antibodies in Chinese individuals infected with HIV-1

**Figure 2:**
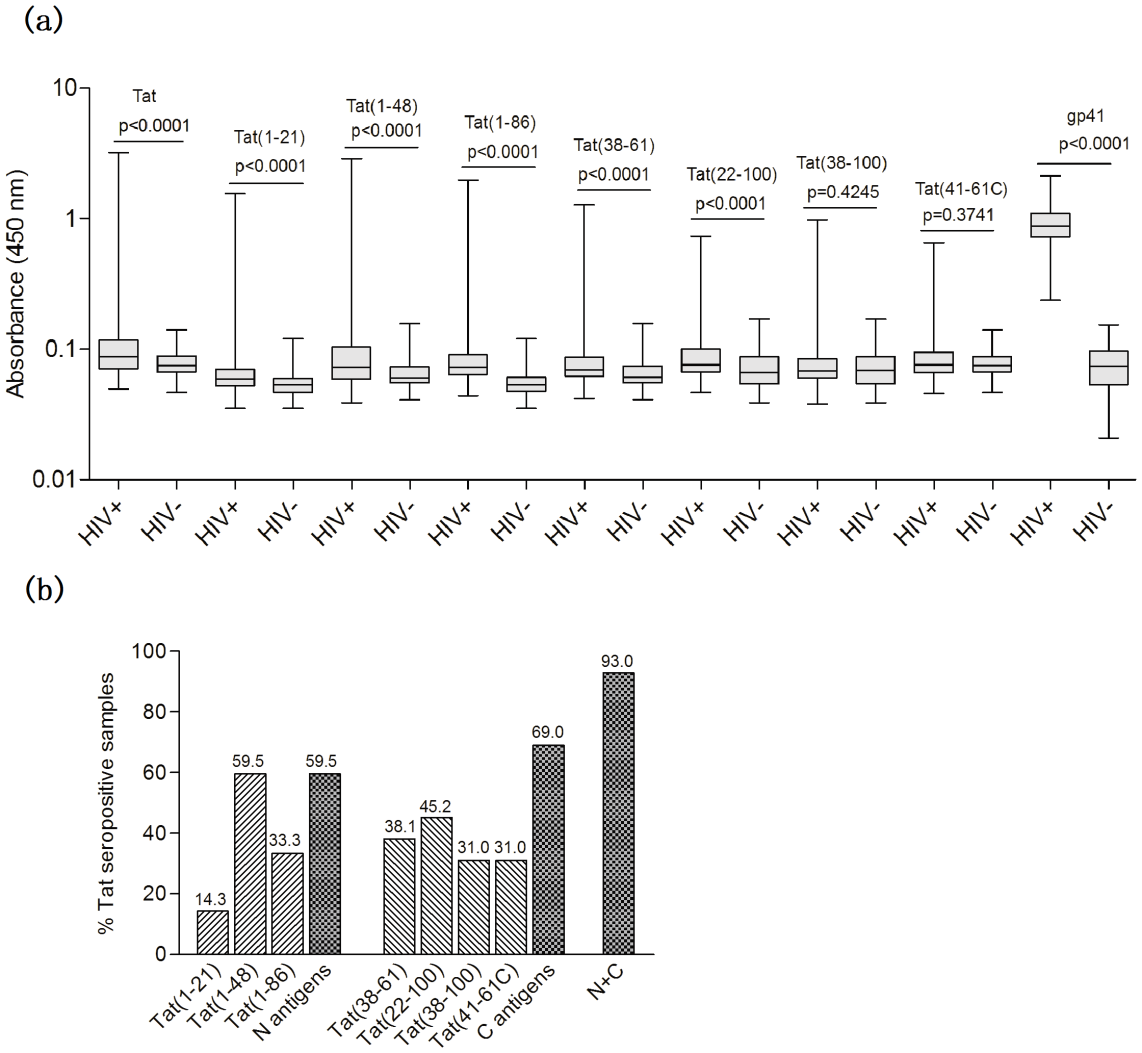
Characterization of antibody responses to full-length Tat and seven Tat peptides. (a) A comparison of the antibody reactivity against Tat of the seven analytic Tat peptides and gp41 in Chinese individual infected with HIV-1. Recombinant subtype B full-length Tat protein and six Tat peptides were used in the assays. The gp41 commercial kit used a pool of peptides representing the immunodominant epitopes in the ectodomain of gp41. The boxes represent the interquartile range, the line inside each box represents the median of the samples and the whiskers represent the range of the data. Statistical significance was tested using Wilcoxon non-parametric test. (b) Characterization of antibody responses to various antigens. The positive rate of antibody response was plotted on the y-axis with the seven antigens on the x-axis. The positive rate corresponding to the N antigens represented the total positive rate of antibody responses detected by Tat(1–21), Tat(1–48) and Tat(1–86) in the 42 anti-Tat-seropositive samples (details were showed in Fig. 3a). The positive rate corresponding to the C antigens represented the total positive rate of antibody responses detected by Tat(38–61), Tat(22–100), Tat(38–100) and Tat(41–61C) in the 42 anti-Tat-seropositive samples. The positive rate (39/42) corresponding to N+C represented the total positive rate of antibody responses detected by the N antigens (25/42) and specific antibody responses only detected by the C antigens (14/42).

We collected 326 HIV-1-infected samples from a clinical cohort of Youan hospital in Beijing, China. Plasma samples from 100 healthy blood donors were included as controls. We established full-length, recombinant, subtype B Tat protein based ELISA assay to screen out Tat-seropositive samples. This assay is able to detect all of the antibody isotypes using HRP-LD5 as conjuate [23], [24]. Out of 326 samples tested, only 42 (12.9%) were positive for anti-Tat antibodies, and most of these (31/42 or 73.8%) showed only weak reactivity (Fig. 2a, 3a). No anti-Tat positive samples were detected in the blood-donor sample. In contrast, gp41 showed strong antigenicity: all 326 samples reacted with gp41, and most of these exhibited either strong or moderate binding reactivity (Fig. 2a).

**Figure 3:**
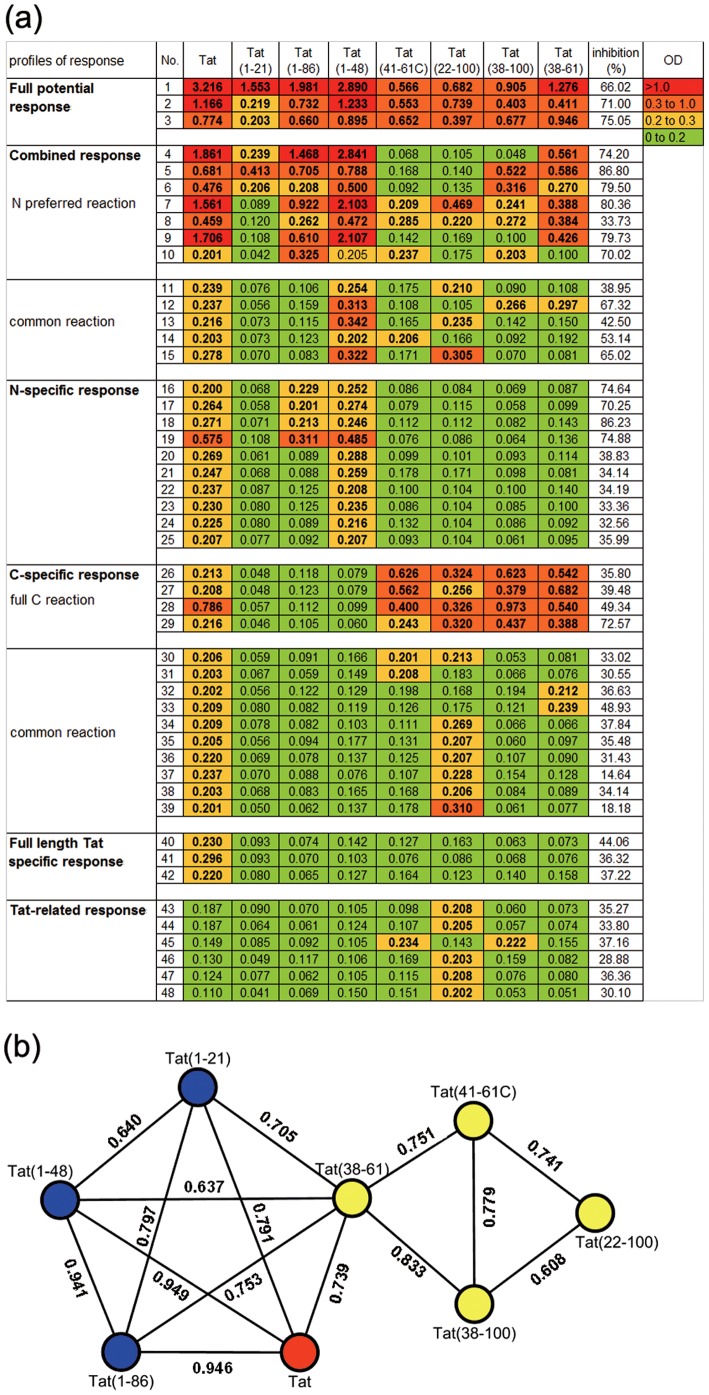
Characteristics of anti-Tat antibody and Tat-neutralizing potential. (a) Characteristics of reactivity with various analytic antigens and the Tat-neutralizing potential of plasma samples of six profiles. The reactivity of each plasma sample with the peptides as assayed by ELISA is presented according to the OD value, as determined by duplicate measurements. Individual values are shown in color, as indicated in the top right corner, with red representing the strongest reaction. The OD values of twelve HIV-1-seropositive and anti-Tat-seronegative plasma samples and 18 plasma samples from healthy blood donors were included as negative controls, which were showed in Table S3. “No.” represents the number of each plasma sample. (b) The correlation analyses between eight analytic antigens based on antibody responses. The correlations with correlation coefficient (R) more than 0.6 and p-value less than 0.05 are shown.

### Characterization of antibody responses to antigens containing various Tat functional domains

In this study, we wished to characterize the anti-Tat responses against different Tat domains using the tailor-made recombinant peptides Tat(1–48), Tat(1–86), Tat(22–100), Tat(38–100), Tat(38–61), Tat(41–61C) and sTat1-21. All of the analytic antigens showed specific binding reactivity to portions of the Tat-seropositive samples with percentages ranging from 13.3–59.5% (Fig. 2b). The positive reactions of each antigen were similar in pattern: i.e., a very small fraction exhibited strong binding reactivity (OD values above 1.0), a small fraction exhibited moderate binding reactivity (OD values between 0.3–1.0) and most exhibited weak binding reactivity (OD values between 0.2–0.3), which suggests a nondominant nature for these antigens (Fig. 3a).

Interestingly, the N-terminal antigen Tat(1–21) or the N-terminus-containing antigens Tat(1–86) and Tat(1–48)—referred to as “N antigens”—showed obviously different reaction patterns compared with antigens lacking the N-terminus—referred to as “C antigens” (Fig. 2b, 3a). The antigenicity of the N antigens showed an obvious gradient: Tat(1–21), Tat(1–86) and Tat(1–48) showed weak, moderate and strong antigenicities, respectively, which reacted with 14.3, 33.3 and 59.5% of the Tat-seropositive samples, respectively (Fig. 2b, 3a). Moreover, all Tat(1–21) positive samples were both Tat(1–48) and Tat(1–86) positive, and all Tat(1–21) or Tat(1–86) positive samples were Tat(1–48) positive (Fig. 3a). This gradient of antigenicity was also present in the reactivity levels (Fig. 3a): all Tat(1–21) samples that exhibited either strong or moderate binding reactivity also exhibited strong or moderate binding reactivity to both Tat(1–48) and Tat(1–86), and all samples that exhibited either strong or moderate binding reactivity to Tat(1–21) or Tat(1–86) also exhibited either strong or moderate binding reactivity to Tat(1–48). Comparing the reactivity patterns of the N antigens to each other as well as to that of full-length Tat, Tat(1–48) and Tat(1–86) showed almost the same reactive patterns, which had a correlation coefficient value (R) of 0.941 (Fig. 3b). To our surprise, both Tat(1–48) and Tat(1–86) had highly similar reactivity patterns compared to full-length Tat, with the R values of 0.946 and 0.949, respectively. This strongly suggests that the important B-cell epitopes of full-length Tat are found in 1–48 aa.

In contrast to the N antigens, the antigenicity of the C antigens showed no obvious gradients. The reactivity rates of the C antigens Tat(22–100), Tat(41–61C), Tat(38–100) and Tat(38–61) with Tat-seropositive samples were 31.0, 45.2, 31.0 and 38.1%, respectively. The total reactivity rate of the C antigens was 69.0% (Fig. 2b). Comparing the reactivity of the C antigens with each other, Tat(22–100)—which contained all domains except the N terminus (1–21)—showed the highest reactivity rate of 45.2%. Unexpectedly, Tat(38–61)—the smallest C antigen—showed a higher reactivity rate (38.1%) than either Tat(41–61C) (31.0%) or Tat(38–100) (31.0%), which both carried at least one more domain than Tat(38–61) (Fig. 1, 2b). Additionally, Tat(41–61C)—which contained an additional basic domain in the N terminus of Tat(22–100)—showed the lowest reactivity rate. These data suggest that the B-cell epitopes in the C antigens are highly conformational and easily affected by surrounding domains.

Other than the obviously different antigenicity, the N and C antigens showed good complementarity for anti-Tat detection. The reactivity rates of the N and C antigens with the Tat-seropositive samples reached 93%, which was much higher than that observed with the N (59.5%) or C antigens (69.0%).

### Characterization of Tat-antibody-response profiles in HIV-1-infected individuals

As described above, both the C and N antigens showed complementary but different reactivity patterns; based on these differences, the anti-Tat responses could be easily classified into one of the following five profile classes (Fig. 3a).

Profile 1) full potential response: Three of the 42 Tat-seropositive samples fell into this category, which was characterized by reactivity, usually strong or moderate, against all of the N and C antigens. All the plasma samples from this profile reacted with full-length Tat at a strong or moderate level.

Profile 2) combined response: Twelve of 42 Tat-seropositive samples fell into this category, which was characterized by reactivity against both N and C antigens. This profile could be further divided into two distinct reaction types: (1) N-preferred reaction, which reacted with both the Tat(1–48) and Tat(1–86) (and possibly more) N antigens as well as with at least one of the C antigens, usually at strong or moderate level. Six of seven plasma samples of this type reacted with full-length Tat at a strong or moderate level. (2) Common reaction, which reacted with one or two of C antigens and only the Tat(1–48) of N antigens at weak or moderate level. All five plasma samples of this type only weakly reacted with full-length Tat.

Profile 3) N-specific response: Ten of 42 Tat-seropositive samples represented the response of this profile which was only characteristically against one, Tat(1–48), or more, Tat(1–86), of the N antigens usually at weak level. The plasma samples with this profile reacted with full-length Tat usually at weak level.

Profile 4) C-specific response: Fourteen of 42 Tat-seropositive samples fell into this category, which was only reactive against the C antigens. This profile, could be further divided into two distinct reaction types: (1) full C reaction, which reacted with all four C antigens, mostly at moderate levels; three of the four plasma samples of this type reacted with full-length Tat at weak level. (2) Common reaction, which reacted with one or more, but not all, of the C antigens at weak level. All ten plasma samples of this type reacted with full-length Tat at weak level.

Profile 5) full-length Tat-specific response: Only three of 42 Tat-seropositive samples fell into this response profile, which was characterized by weak reactivity against full-length Tat, but no reactivity against the N and C antigens.

Considering the nonimmunodominant nature of Tat, we screened out 6 samples with the highest anti-Tat OD values from 100 HIV-seropositive and anti-Tat-seronegative samples and further assessed their reactivities with the N and C antigens, and we uncovered yet another response profile. Profile 6) Tat-related response: Six of the 100 Tat-seronegative samples fell into this category, which was characterized by reactivity against C antigens at weak level (Fig. 3a). It was also very interesting to find that five of these six samples reacted with Tat(22–100).

### Characterization of the Tat-neutralization potential of the different response profiles

**Figure 4:**
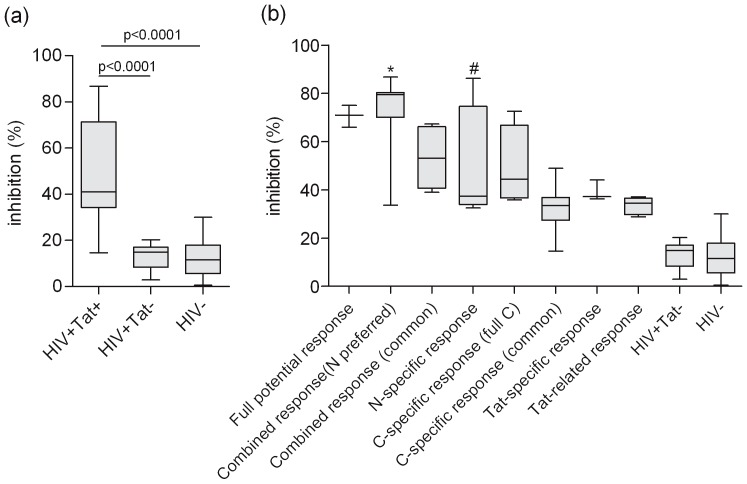
Comparison of Tat-neutralizing potential of plasma samples. (a) Antibody-mediated neutralization of exogenous recombinant full-length Tat. The percent inhibition of transactivation at 48 h was plotted on the y-axis with the following samples on the x-axis: HIV+Tat+, HIV-1-seropositive and anti-Tat-seropositive plasma; HIV+Tat-, HIV-1-seropositive and anti-Tat-seronegative plasma; and HIV-, healthy blood-donor plasma. The data on the y-axis represent the inhibition of the SEAP release in the culture supernatant of HEK293T cells, as described in the “Materials and Methods” section. The boxes represent the interquartile range, the line inside each box represents the median of the samples and the whiskers represent the range of the data. Statistical significance was tested using Wilcoxon non-parametric test. (b) A comparison of eight Tat-antibody reactive types in the six response profiles and percent inhibition of transactivation. The percent inhibition at 48 h was plotted on the y-axis with samples classified by Tat antibody response profile on the x-axis. Statistical significance was tested using Nemenyi non-parametric test. * p < 0.05 comparing HIV+Tat- and HIV- groups. ^#^p < 0.05 comparing HIV- group.

Forty-eight samples from these six profiles, twelve anti-Tat-negative HIV samples and 18 healthy blood-donor samples were evaluated for extracellular Tat-neutralization activity. The percentage of SEAP-expression inhibition for each group is presented in Fig. 4a. Anti-Tat-positive samples showed significantly higher Tat-neutralizing activities comparing with anti-Tat-negative and blood-donor samples (Fig. 4a). Among the six immunological profiles, the N-preferred reaction in combined response showed significant Tat-neutralizing activity (Fig. 4b), which was significantly higher compared with the HIV-1-seropositive and anti-Tat-seronegative group (HIV+Tat-) and healthy blood-donor plasma (HIV-) group. We choose ten samples with higher antibody reactivity and neutralizing activity to further assess the neutralization activity after depleting the anti-Tat antibodies or IgG fractions of the plasma. These samples lost entire and most neutralization activity after depleting the anti-Tat antibodies or IgG fraction with about 20% inhibition of transactivation ability, which is similar to the percent inhibition from HIV+Tat- and HIV- plasma samples (Table 1). These demonstrated that anti-Tat antibodies are specifically responsible for the neutralization activity and IgG fraction contributes to most of this neutralization activity.

**Table 1:** Percent inhibition of Tat-transactivation after depleting anti-Tat antibodies and IgG fractions of the plasma.

profiles of response	No.	Tat	inhibition (%)^a^	inhibition (%)^b^	inhibition (%)^c^
Full potential response	1	3.216	74.01	27.59	43.85
	2	1.166	76.83	30.87	39.63
	3	0.774	80.95	13.68	20.54
Combined response(N preferred reaction)	4	1.861	70.36	38.43	47.42
	5	0.681	82.45	22.74	36.72
	6	0.476	78.60	17.25	15.37
	7	1.561	74.54	26.36	37.31
	9	1.706	72.19	19.14	38.95
N-specific response	17	0.264	70.28	11.31	17.29
	18	0.271	68.27	16.95	13.28
					
monoclonal Tat antibody		2.518	75.25	12.58	11.95
					
HIV+Tat-	1	0.065	22.55	20.12	18.70
	2	0.06	18.18	15.20	19.12
	3	0.051	9.41	8.57	7.31
	4	0.053	26.37	19.26	22.12
	5	0.058	17.66	15.33	13.15
	6	0.053	7.33	10.02	9.87
	7	0.054	15.79	11.78	9.96
	8	0.067	23.28	12.12	17.83
	9	0.063	13.75	14.00	12.26
	10	0.062	12.65	10.98	12.82
					
HIV-	1	0.087	11.47	12.42	13.16
	2	0.076	18.59	10.78	17.89
	3	0.058	23.43	18.72	21.10
	4	0.071	8.39	10.80	7.89
	5	0.072	19.34	16.54	13.32
	6	0.071	3.23	5.33	7.88
	7	0.083	15.55	10.40	7.97
	8	0.064	24.93	15.87	16.89
	9	0.087	16.72	17.80	14.38
	10	0.067	9.78	11.20	7.72

All sera were diluted at 1:50 for Tat-neutralization assay. The ELISA OD values and inhibition of Tat-transactivation (inhibition (%)) are shown. “No.” represents the number of each plasma sample.^ a^ % inhibition of Tat-transactivation after depleting non-specific Tat antibodies with coated pPEPTIDE2 by ELISA.^ b^ % inhibition of Tat-transactivation after depleting Tat antibodies with coated Tat by ELISA.^ c^ % inhibition of Tat-transactivation after depleting IgG fractions with coated protein G by ELISA.

**Figure 5:**
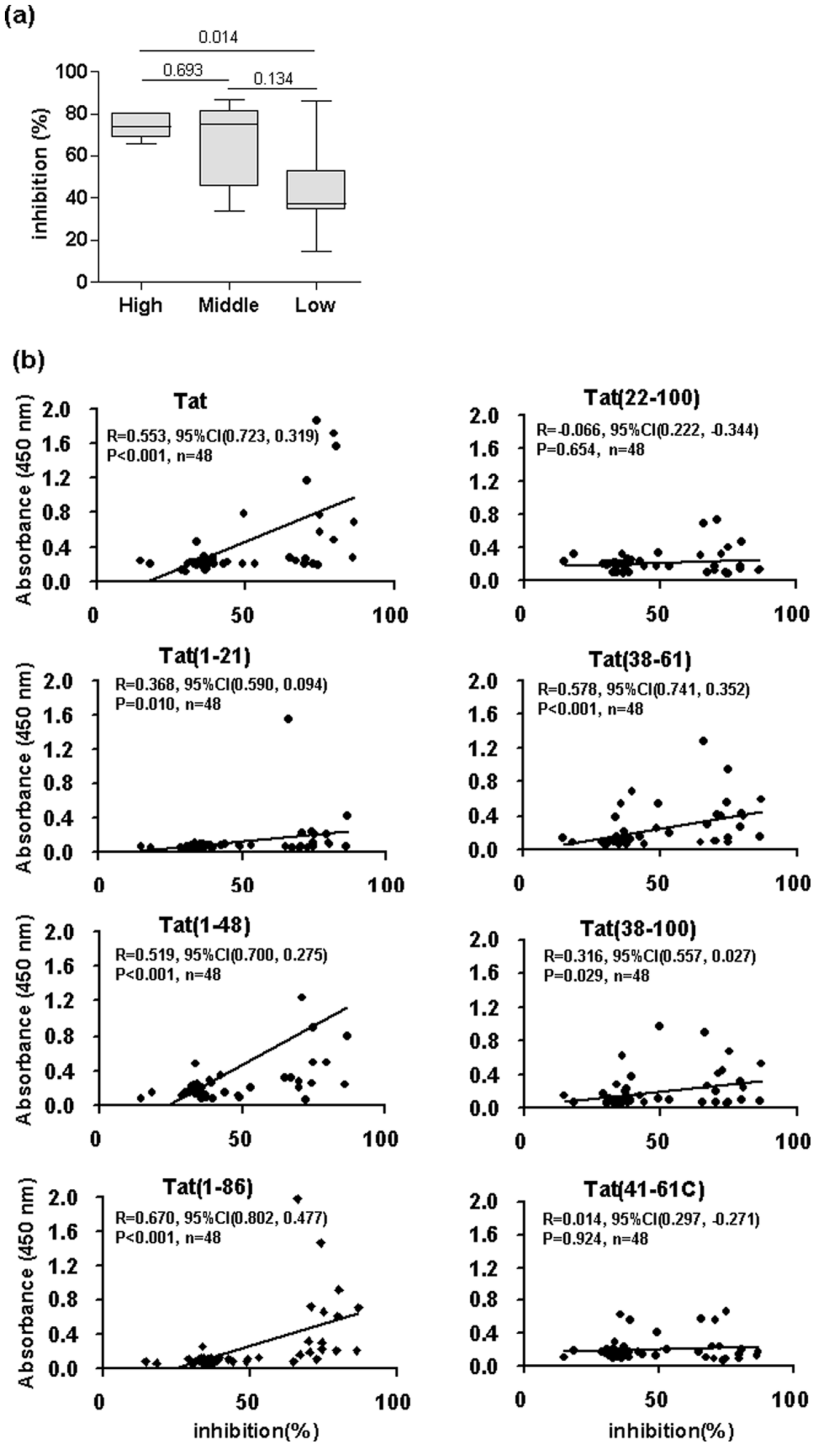
The correlations of antibody reactivity against eight antigens and percent inhibition of transactivation. (a) Antibody-mediated neutralization of exogenous recombinant full-length Tat. Percent inhibition at 48 h was plotted on the y-axis with samples classified by Tat-antibody levels on the x-axis (High—samples with strong reactivity to full-length Tat with OD values above 1.0; Middle—samples with moderate reactivity to full-length Tat with OD values between 0.3–1.0; Low—samples with weak reactivity to full-length Tat with OD values between 0.2–0.3). Statistical significance was tested using Wilcoxon non-parametric test. (b) A comparison of the correlations of antibody reactivity against full-length Tat or the analytic antigens and percent inhibition of transactivation. The correlation was assessed by Spearman correlation coefficient. Correlation coefficient values (R), p values and the samples size (n) are shown.

Statistical analyses revealed that the neutralizing activity of the group that exhibited strong binding reactivity (OD values above 1.0) to full-length Tat was significantly higher than the group that exhibited weak binding reactivity (OD values between 0.2–0,3) (Fig. 5a). Correlation analyses between the antibody reactivity of each antigen and Tat-neutralizing activity were carried out for the 48 samples from these six profiles. We found that the reactivity with Tat(1–86), Tat(1–48), full-length Tat, Tat(38–61), Tat(38–100) and Tat(1–21) was significantly correlated with Tat-neutralizing activity (Fig. 5b).

## Discussion

In this study, we define for the first time host’s anti-Tat responses in Chinese patients infected with HIV-1. Consistent with the previous findings that Tat is intrinsically nonimmunodominant in nature [13], [14], [15], [16], [17], our results also verified this conclusion (Fig. 2). This nonimmunodominant property is consistent with the results of structural studies which revealed that HIV Tat is an intrinsically unstructured protein, or unfolded protein lack of secondary structures and high structures [28], [29], [30], [31], and which could be likely to help avoid elicting the host’s anti-Tat immunity.

In this study, the N antigens and C antigens clearly showed obviously different antigenicity (Fig. 2b, 3). Moreover, there were still some differences in antigenicity between different N antigens or different C antigens (Fig. 2b, 3). Based on these differences, we characterized six immunological profiles: full potential response, combined response, N-specific response, C-specific response, full-length Tat-specific response and Tat-related response. Interestingly, samples from the full-potential response and N-preferred reaction type in the combined response profile presented strong or moderate reactions to the N, C and full-length Tat antigens and also showed the highest neutralizing activity (Fig. 3, 4). These sample types only accounted for 23.8% of total Tat-seropositive samples, and they represent the strongest reactive groups. In contrast, all other samples (except for the samples of full C reaction type in C specific response profile) almostly present weak reactions to N, C and full-length Tat antigens, and showed weak neutralizing activity (Fig. 3, 4). Based on these findings and the fact that Tat is intrinsically nonimmunodominant, we hypothesize that the full-potential response profile and N-preferred reaction in combined response profile represent the major complete anti-Tat antibody response, which was elicited recently by a large amount of transiently released Tat produced on the occasion of vigorous replication of the newly immuno-escaped HIV strains, and target all, or at least multiple, N and C epitopes of Tat. It is possible that the other response profiles (except for the full C reaction type and part of the common reaction type in C-specific response profile) could represent various degraded forms of this complete antibody response. Follow up of these Tat-seropositive individuals will help to verify this hypothesis.

It was also interesting to find that the samples from the full C reaction in C-specific response profile showed moderate reactivity to all four C antigens but weak reactivity to full-length Tat (Fig. 3), as well as showed significant neutralizing activity (Fig. 4). This result could suggest that there were few individuals (∼9.5%) who elicited an alternative antibody response that is more specific to C antigens (C-prone response) other than the major complete anti-Tat antibody response. Some samples of the common reaction type in C-specific response profile might represent the degraded form of this C-prone response. The possibility that these subjects are genetically predisposed to the C-prone response needs to be evaluated in future studies.

Very interesting, Tat(1–21) had previously been shown to be a dominant linear epitope. In addition to the Tat(1–21) domain, the Tat(1–48) antigen contains an additional cysteine-rich domain (CRD), which was recently identified as an immunodominant B-cell epitope [27] and could account for the enhanced antigenicity of Tat(1-48). Unexpectedly, Tat(1-86), which contained at least one more known epitope (the BD epitope) in addition to those in Tat(1–48), showed weaker antigenicity compared with Tat(1–48). This indicates that the 49–86 aa in Tat(1–86) does not provide additional reactive epitopes but does affect the conformation of the reactive epitopes found in the Tat(1–48) fragment, which provides new supportive evidence for the conformational nature of the Tat antigen [32].

Theoretically, Tat-neutralization potential, as opposed to anti-Tat reactivity, could represent well the anti-Tat protective role. Our study shows that strong Tat-neutralization potential is related to N-preferred reaction in combined response immunoprofiles which represent newly elicited complete anti-Tat antibody responses (Fig. 4b). Also, Tat-neutralization potential was showed to be significantly related to the reactivity of the specific antigens Tat(1–86), Tat(38–61), full-length Tat, Tat(1–48), Tat(1–21) and Tat(38–100). Among N antigens, the reactivity of Tat(1–48) and Tat(1–86) significantly related to Tat-neutralization potential. Considering that the reactivity of Tat(1–48) and Tat(1–86) is highly correlated with that of full-length Tat (Fig. 3b), and the previous findings that B-clade Tat(1–86) is the mostly well-characterized active form of Tat, it is reasonable to conclude that the principle protective epitopes of full-length Tat should be in the N-terminal 1-48 aa. Among four C antigens, only the reactivity of Tat(38–61) and Tat(38–100) significantly related to Tat-neutralization potential (Fig. 4b). It was intriguing to find that the reactivity of Tat(38-100) showed a highest correlation to that of Tat(38–61) with R value of 0.833 among all C antigens. Considering that all four of the C antigens contained the 38–61 aa domain, Tat(38–61) and Tat(38–100) carried no or the least additional amino acids which could affect the conformation of Tat(38–61), it is reasonable to hypothesize that Tat(38–61) contains an another important protective epitope. These findings are likely to be important for understanding of protective anti-Tat antibody responses as well as for future vaccine designs.

In conclusion, in this study, we define for the first time six different immunoprofiles of anti-Tat responses in Chinese patients infected with HIV-1, which represent two types of anti-Tat antibody responses: the major complete response and the alternative C-prone response. Tat-neutralizing potential was demonstrated to be significantly related to specific immunoprofiles and to the reactivity of specific antigens. The findings presented here could significantly contribute to our understanding of anti-Tat responses in preventing Tat-mediated HIV pathogenesis and aid in future vaccine designs.

## Supporting Information

Table S1 **Baseline characteristics of the study participants.**
(DOC)

Table S2 **Primers for amplifying HIV-1 Tat peptides.**
(DOC)

Table S3 **Tat anti-sera reactivity to Tat peptides.**
(DOC)
