# Supplementary material for: Characterization of Tat Antibody Responses in Chinese Individuals Infected with HIV-1
Source: PLoS One. 2013 Apr 2;8(4):e60825. doi: 10.1371/journal.pone.0060825 (PMC3614898; doi:10.1371/journal.pone.0060825)
Supplement: Table S1 — Baseline characteristics of the study participants. (DOC) [file pone.0060825.s001.doc]

|  | (n=326)^a^ |
| --- | --- |
| **Age (yr)** |  |
| Mean ± s.d.^b^ | 34.7±8.4 |
| Range | 13-59 |
| **Sex (%)** |  |
| Male | 77.3 |
| Female | 22.7 |
| **CD4^+^ (cells/µl )** |  |
| Mean ± s.d. | 429.3±146.6 |
| Range | 11-1144 |
| **CD4^+^/CD8^+^ (%)** |  |
| ＜0.5 | 73.0 |
| **Transmission (%)** |  |
| sex transmission | 75.4 |
| blood transmission | 24.3 |
| vertical transmission | 0.3 |
| **Time since HAART initiation (yr)^c^** |  |
| Mean ± s.d. | 5±2 |
| Range | 1-13 |

^a^Number of evaluable individuals; ^b^Standard deviation; ^c^Based on 145 individuals, the rest of 181 individuals were drug naive.
