# Supplementary material for: Characterization of Tat Antibody Responses in Chinese Individuals Infected with HIV-1
Source: PLoS One. 2013 Apr 2;8(4):e60825. doi: 10.1371/journal.pone.0060825 (PMC3614898; doi:10.1371/journal.pone.0060825)
Supplement: Table S2 — Primers for amplifying HIV-1 Tat peptides. (DOC) [file pone.0060825.s002.doc]

| Primers | Sequences (5'-3') | Description |
| --- | --- | --- |
| U1 | CGCCCGCGGATTAATGGCATGGAACCGGTTGAC | The primer pairs U1/D48, U1/D86, U22/D100, U38/D100 and U38/D61 were used to amplify Tat(1-48), Tat(1-86), Tat(22-100), Tat(38-100) and Tat(38-61), respectively. U1, U22 and U38 contain AseI restriction sites, and D100 contains a BamHI restriction site (underlined) |
| D48 | GGCCCGGGGATCCTAACCGTAAGAGATACCCAG |  |
| D86 | GGCCCGGGGATCCTATTCTTTCGGACCGGTCG |  |
| U22 | CGCCCGCGGATTAATGGCTGCACCAACTGCTAC |  |
| U38 | CGCCCGCGGATTAATGGCTTCATCACCAAAGCT |  |
| D100 | GGCCCGGGGATCCTAGTCGAACGGGTCGGTTTC |  |
| D61 | GGCCCGGGGATCCTAACCCTGCGGCGGACGAC |  |
| U41 | CAAGGCCATGGCTGATATCATTAATGGCAAAGCTCTGGGTATCTCTTA | U41 and D41-61C were used to amplify Tat(41-61), UC and D100P were used to amplify Tat(22-100), U41 contains AseI restriction sites (underlined), and D100P contains a BamHI restriction site (underlined) |
|  |  |  |
| D41-61C | AGTAGCAGTTGGTGCAGTTCTGGTGAGCACGACGAC |  |
| UC | TGCACCAACTGCTACTGCAA |  |
| D100P | TCGAGTGCGGCCGCGGATCCTAGTCGAACGGGTCGGTTTCGG |  |
|  |  | Upet and Dpet contain AseI and BamHI restriction sites, respectively, (underlined) and were used to amplify Tat(41-61C) by overlapping PCR |
| Upet | CAAGGCCATGGCTGATATCCGCCCGCGGATTAATGGC |  |
| Dpet | GCTCGAGTGCGGCCGCGGATCCTA |  |
