# Supplementary material for: Characterization of Tat Antibody Responses in Chinese Individuals Infected with HIV-1
Source: PLoS One. 2013 Apr 2;8(4):e60825. doi: 10.1371/journal.pone.0060825 (PMC3614898; doi:10.1371/journal.pone.0060825)
Supplement: Table S3 — Tat anti-sera reactivity to Tat peptides. (DOC) [file pone.0060825.s003.doc]

| profiles of response | No. | Tat | Tat (1-21) | Tat (1-86) | Tat (1-48) | Tat (41-61C) | Tat (22-100) | Tat (38-100) | Tat (38-61) | inhibition (%) | CD4^+^  (cells/µl) | CD8^+^  (cells/µl) | CD4^+^/CD8^+^ | viral load (copies/ml) |
| --- | --- | --- | --- | --- | --- | --- | --- | --- | --- | --- | --- | --- | --- | --- |
| Full potential response | 1 | **3.216** | **1.553** | **1.981** | **2.890** | **0.566** | **0.682** | **0.905** | **1.276** | 66.02 | 414 | 1317 | 0.31 | 66000 |
|  | 2 | **1.166** | **0.219** | **0.732** | **1.233** | **0.553** | **0.739** | **0.403** | **0.411** | 71.00 | 202 | 933 | 0.22 | - |
|  | 3 | **0.774** | **0.203** | **0.660** | **0.895** | **0.652** | **0.397** | **0.677** | **0.946** | 75.05 | 591 | 1305 | 0.45 | 12000 |
|  |  |  |  |  |  |  |  |  |  |  |  |  |  |  |
| Combined response (N preferred reaction) | 4 | **1.861** | **0.239** | **1.468** | **2.841** | 0.068 | 0.105 | 0.048 | **0.561** | 74.20 | 389 | 1265 | 0.31 | 37967 |
|  | 5 | **0.681** | **0.413** | **0.705** | **0.788** | 0.168 | 0.140 | **0.522** | **0.586** | 86.80 | 245 | 959 | 0.26 | 173000 |
|  | 6 | **0.476** | **0.206** | **0.208** | **0.500** | 0.092 | 0.135 | **0.316** | **0.270** | 79.50 | 108 | 716 | 0.15 | 85070 |
|  | 7 | **1.561** | 0.089 | **0.922** | **2.103** | **0.209** | **0.469** | **0.241** | **0.388** | 80.36 | – | – | – | 17000 |
|  | 8 | **0.459** | 0.120 | **0.262** | **0.472** | **0.285** | **0.220** | **0.272** | **0.384** | 33.73 | 441 | 673 | 0.66 | 13000 |
|  | 9 | **1.706** | 0.108 | **0.610** | **2.107** | 0.142 | 0.169 | 0.100 | **0.426** | 79.73 | – | – | – | 8000 |
|  | 10 | **0.201** | 0.042 | **0.325** | 0.205 | **0.237** | 0.175 | **0.203** | 0.100 | 70.02 | 247 | 998 | 0.25 | - |
|  |  |  |  |  |  |  |  |  |  |  |  |  |  |  |
| Combined response (common reaction) | 11 | **0.239** | 0.076 | 0.106 | **0.254** | 0.175 | **0.210** | 0.090 | 0.108 | 38.95 | – | – | – | 200000 |
|  | 12 | **0.237** | 0.056 | 0.159 | **0.313** | 0.108 | 0.105 | **0.266** | **0.297** | 67.32 | 514 | 1911 | 0.27 | 840 |
|  | 13 | **0.216** | 0.073 | 0.115 | **0.342** | 0.165 | **0.235** | 0.142 | 0.150 | 42.50 | 482 | 1352 | 0.36 | 4200 |
|  | 14 | **0.203** | 0.073 | 0.123 | **0.202** | **0.206** | 0.166 | 0.092 | 0.192 | 53.14 | 634 | 927 | 0.68 | 460 |
|  | 15 | **0.278** | 0.070 | 0.083 | **0.322** | 0.171 | **0.305** | 0.070 | 0.081 | 65.02 | 312 | 964 | 0.32 | - |
|  |  |  |  |  |  |  |  |  |  |  |  |  |  |  |
| N-specific response | 16 | **0.200** | 0.068 | **0.229** | **0.252** | 0.086 | 0.084 | 0.069 | 0.087 | 74.64 | 698 | 2636 | 0.26 | 11000 |
|  | 17 | **0.264** | 0.058 | **0.201** | **0.274** | 0.079 | 0.115 | 0.058 | 0.099 | 70.25 | 211 | 1183 | 0.18 | 1500 |
|  | 18 | **0.271** | 0.071 | **0.213** | **0.246** | 0.112 | 0.112 | 0.082 | 0.143 | 86.23 | 298 | 709 | 0.42 | 103000 |
|  | 19 | **0.575** | 0.108 | **0.311** | **0.485** | 0.076 | 0.086 | 0.064 | 0.136 | 44.52 | 299 | 1301 | 0.23 | 210000 |
|  | 20 | **0.269** | 0.061 | 0.089 | **0.288** | 0.099 | 0.101 | 0.093 | 0.114 | 38.83 | 284 | 964 | 0.29 | 4800 |
|  | 21 | **0.247** | 0.068 | 0.088 | **0.259** | 0.178 | 0.171 | 0.098 | 0.081 | 34.14 | 513 | 1210 | 0.42 | 5600 |
|  | 22 | **0.237** | 0.087 | 0.125 | **0.208** | 0.100 | 0.104 | 0.100 | 0.140 | 34.19 | 526 | 1048 | 0.50 | - |
|  | 23 | **0.230** | 0.080 | 0.125 | **0.235** | 0.086 | 0.104 | 0.085 | 0.100 | 33.36 | 527 | 892 | 0.59 | 4200 |
|  | 24 | **0.225** | 0.080 | 0.089 | **0.216** | 0.132 | 0.104 | 0.086 | 0.092 | 32.56 | 663 | 863 | 0.77 | 2300 |
|  | 25 | **0.207** | 0.077 | 0.092 | **0.207** | 0.093 | 0.104 | 0.061 | 0.095 | 35.99 | 471 | 974 | 0.48 | 46853 |
|  |  |  |  |  |  |  |  |  |  |  |  |  |  |  |
| C-specific response (full C reaction) | 26 | **0.213** | 0.048 | 0.118 | 0.079 | **0.626** | **0.324** | **0.623** | **0.542** | 35.80 | 412 | 1162 | 0.35 | - |
|  | 27 | **0.208** | 0.048 | 0.123 | 0.079 | **0.562** | **0.256** | **0.379** | **0.682** | 39.48 | 219 | 942 | 0.23 | 160000 |
|  | 28 | **0.786** | 0.057 | 0.112 | 0.099 | **0.400** | **0.326** | **0.973** | **0.540** | 49.34 | 301 | 804 | 0.37 | - |
|  | 29 | **0.216** | 0.046 | 0.105 | 0.060 | **0.243** | **0.320** | **0.437** | **0.388** | 72.57 | 1058 | 730 | 1.45 | 1700 |
|  |  |  |  |  |  |  |  |  |  |  |  |  |  |  |
| C-specific response (common reaction) | 30 | **0.206** | 0.059 | 0.091 | 0.166 | **0.201** | **0.213** | 0.053 | 0.081 | 33.02 | – | – | – | - |
|  | 31 | **0.203** | 0.067 | 0.059 | 0.149 | **0.208** | 0.183 | 0.066 | 0.076 | 30.55 | 483 | 891 | 0.54 | - |
|  | 32 | **0.202** | 0.056 | 0.122 | 0.129 | 0.198 | 0.168 | 0.194 | **0.212** | 36.63 | - | - | - | 43500 |
|  | 33 | **0.209** | 0.080 | 0.082 | 0.119 | 0.126 | 0.175 | 0.121 | **0.239** | 48.93 | 181 | 782 | 0.23 | 250 |
|  | 34 | **0.209** | 0.078 | 0.082 | 0.103 | 0.111 | **0.269** | 0.066 | 0.066 | 37.84 | 282 | 1492 | 0.19 | - |
|  | 35 | **0.205** | 0.056 | 0.094 | 0.177 | 0.131 | **0.207** | 0.060 | 0.097 | 35.48 | 298 | 1041 | 0.29 | - |
|  | 36 | **0.220** | 0.069 | 0.078 | 0.137 | 0.125 | **0.207** | 0.107 | 0.090 | 31.43 | – | – | – | - |
|  | 37 | **0.237** | 0.070 | 0.088 | 0.076 | 0.107 | **0.228** | 0.154 | 0.128 | 14.64 | 136 | 199 | 0.68 | LDL |
|  | 38 | **0.203** | 0.068 | 0.083 | 0.165 | 0.168 | **0.206** | 0.084 | 0.089 | 34.14 | 513 | 1210 | 0.42 | 5600 |
|  | 39 | **0.201** | 0.050 | 0.062 | 0.137 | 0.178 | **0.310** | 0.061 | 0.077 | 18.18 | 245 | 557 | 0.44 | - |
|  |  |  |  |  |  |  |  |  |  |  |  |  |  |  |
| Full length Tat specific response | 40 | **0.230** | 0.093 | 0.074 | 0.142 | 0.127 | 0.163 | 0.063 | 0.073 | 44.06 | 239 | 765 | 0.31 | - |
|  | 41 | **0.296** | 0.093 | 0.070 | 0.103 | 0.076 | 0.086 | 0.068 | 0.076 | 36.32 | 133 | 730 | 0.18 | - |
|  | 42 | **0.220** | 0.080 | 0.065 | 0.127 | 0.164 | 0.123 | 0.140 | 0.158 | 37.22 | 470 | 802 | 0.59 | 991 |
|  |  |  |  |  |  |  |  |  |  |  |  |  |  |  |
| Tat-related response | 43 | 0.187 | 0.090 | 0.070 | 0.105 | 0.098 | **0.208** | 0.060 | 0.073 | 35.27 | 338 | 1538 | 0.22 | - |
|  | 44 | 0.187 | 0.064 | 0.061 | 0.124 | 0.107 | **0.205** | 0.057 | 0.074 | 33.80 | 342 | 897 | 0.38 | - |
|  | 45 | 0.149 | 0.085 | 0.092 | 0.105 | **0.234** | 0.143 | **0.222** | 0.155 | 37.16 | 642 | 1383 | 0.46 | - |
|  | 46 | 0.130 | 0.049 | 0.117 | 0.106 | 0.169 | **0.203** | 0.159 | 0.082 | 28.88 | – | – | – | 200000 |
|  | 47 | 0.124 | 0.077 | 0.062 | 0.105 | 0.115 | **0.208** | 0.076 | 0.080 | 36.36 | 321 | 864 | 0.37 | - |
|  | 48 | 0.110 | 0.041 | 0.069 | 0.150 | 0.151 | **0.202** | 0.053 | 0.051 | 30.10 | 405 | 1074 | 0.38 | - |
|  |  |  |  |  |  |  |  |  |  |  |  |  |  |  |
| HIV+Tat- | 1 | 0.065 | 0.047 | 0.055 | 0.071 | 0.087 | 0.091 | 0.058 | 0.051 | 10.20 | – | – | – | LDL |
|  | 2 | 0.06 | 0.037 | 0.052 | 0.059 | 0.063 | 0.065 | 0.039 | 0.042 | 2.93 | – | – | – | 10000 |
|  | 3 | 0.051 | 0.05 | 0.05 | 0.063 | 0.065 | 0.086 | 0.045 | 0.06 | 7.82 | – | – | – | LDL |
|  | 4 | 0.053 | 0.049 | 0.038 | 0.053 | 0.065 | 0.077 | 0.05 | 0.051 | 16.25 | – | – | – | 12300 |
|  | 5 | 0.058 | 0.037 | 0.049 | 0.059 | 0.064 | 0.069 | 0.048 | 0.054 | 18.05 | – | – | – | 19100 |
|  | 6 | 0.053 | 0.044 | 0.043 | 0.051 | 0.063 | 0.076 | 0.047 | 0.059 | 3.01 | – | – | – | 41600 |
|  | 7 | 0.054 | 0.043 | 0.057 | 0.05 | 0.071 | 0.08 | 0.047 | 0.047 | 12.19 | – | – | – | - |
|  | 8 | 0.067 | 0.048 | 0.059 | 0.058 | 0.062 | 0.07 | 0.056 | 0.052 | 16.43 | – | – | – | 8400 |
|  | 9 | 0.063 | 0.048 | 0.044 | 0.058 | 0.068 | 0.066 | 0.054 | 0.054 | 20.25 | – | – | – | - |
|  | 10 | 0.062 | 0.043 | 0.061 | 0.054 | 0.061 | 0.061 | 0.055 | 0.046 | 17.20 | – | – | – | 13000 |
|  | 11 | 0.062 | 0.048 | 0.063 | 0.058 | 0.064 | 0.068 | 0.056 | 0.057 | 14.35 | – | – | – | 4070 |
|  | 12 | 0.072 | 0.049 | 0.082 | 0.078 | 0.064 | 0.071 | 0.085 | 0.079 | 15.49 | – | – | – | 4360 |
|  |  |  |  |  |  |  |  |  |  |  |  |  |  |  |
| HIV- | 1 | 0.087 | 0.052 | 0.059 | 0.069 | 0.073 | 0.11 | 0.052 | 0.059 | 13.39 | – | – | – | – |
|  | 2 | 0.076 | 0.042 | 0.056 | 0.057 | 0.068 | 0.075 | 0.054 | 0.052 | 11.77 | – | – | – | – |
|  | 3 | 0.058 | 0.048 | 0.054 | 0.055 | 0.071 | 0.072 | 0.052 | 0.056 | 30.00 | – | – | – | – |
|  | 4 | 0.071 | 0.049 | 0.049 | 0.062 | 0.097 | 0.083 | 0.054 | 0.053 | 22.56 | – | – | – | – |
|  | 5 | 0.072 | 0.051 | 0.06 | 0.08 | 0.114 | 0.111 | 0.055 | 0.059 | 22.92 | – | – | – | – |
|  | 6 | 0.071 | 0.056 | 0.063 | 0.075 | 0.086 | 0.09 | 0.057 | 0.06 | 19.15 | – | – | – | – |
|  | 7 | 0.083 | 0.05 | 0.057 | 0.072 | 0.079 | 0.087 | 0.056 | 0.061 | 17.53 | – | – | – | – |
|  | 8 | 0.064 | 0.049 | 0.067 | 0.067 | 0.067 | 0.078 | 0.056 | 0.057 | 14.76 | – | – | – | – |
|  | 9 | 0.087 | 0.046 | 0.064 | 0.1 | 0.119 | 0.161 | 0.068 | 0.057 | 11.23 | – | – | – | – |
|  | 10 | 0.067 | 0.061 | 0.07 | 0.087 | 0.077 | 0.086 | 0.071 | 0.065 | 10.88 | – | – | – | – |
|  | 11 | 0.065 | 0.045 | 0.068 | 0.061 | 0.074 | 0.074 | 0.055 | 0.053 | 8.27 | – | – | – | – |
|  | 12 | 0.064 | 0.045 | 0.065 | 0.06 | 0.067 | 0.063 | 0.068 | 0.06 | 6.77 | – | – | – | – |
|  | 13 | 0.088 | 0.051 | 0.066 | 0.085 | 0.079 | 0.118 | 0.059 | 0.068 | 3.90 | – | – | – | – |
|  | 14 | 0.097 | 0.056 | 0.09 | 0.12 | 0.077 | 0.087 | 0.107 | 0.076 | 3.33 | – | – | – | – |
|  | 15 | 0.09 | 0.053 | 0.048 | 0.074 | 0.076 | 0.071 | 0.051 | 0.051 | 0.52 | – | – | – | – |
|  | 16 | 0.114 | 0.055 | 0.054 | 0.088 | 0.087 | 0.07 | 0.048 | 0.05 | 2.75 | – | – | – | – |
|  | 17 | 0.073 | 0.036 | 0.065 | 0.07 | 0.07 | 0.067 | 0.061 | 0.057 | 6.16 | – | – | – | – |
|  | 18 | 0.14 | 0.039 | 0.058 | 0.153 | 0.129 | 0.119 | 0.05 | 0.066 | 15.11 | – | – | – | – |

All sera were diluted at 1:10 for ELISA and 1:50 for Tat-neutralization assay. The ELISA OD values, inhibition of Tat-transactivation (inhibition (%)), CD4^+^, CD8^+^ T cell counts and viral load are shown. “No.” represents the number of each plasma sample. “LDL” represents viral load below the lower detection limit. “–” represents not detected.
